# 18-Fluoro-2-deoxy-d-glucose positron emission tomography/computed tomography scan for monitoring the therapeutic response in experimental *Staphylococcus aureus* foreign-body osteomyelitis

**DOI:** 10.1186/s13018-015-0274-9

**Published:** 2015-08-27

**Authors:** Sofia Chatziioannou, Odysseas Papamichos, Maria N. Gamaletsou, Alexandros Georgakopoulos, Nikolaos G. Kostomitsopoulos, Sofia Tseleni-Balafouta, Joseph Papaparaskevas, Thomas J. Walsh, Spiros G. Pneumaticos, Nikolaos V. Sipsas

**Affiliations:** Second Department of Radiology, Medical School, National and Kapodistrian University of Athens, Athens, Greece; Department of Pathophysiology, Medical School, National and Kapodistrian University of Athens, Mikras Asias 75, Athens, 115 27 Greece; Third Department of Orthopedics, Medical School, National and Kapodistrian University of Athens, Athens, Greece; Department of Pathology, Medical School, National and Kapodistrian University of Athens, Athens, Greece; Department of Microbiology, Medical School, National and Kapodistrian University of Athens, Athens, Greece; PET/CT Section, Foundation for Biomedical Research of the Academy of Athens, Athens, Greece; Center for Experimental Surgery, Foundation for Biomedical Research of the Academy of Athens, Athens, Greece; Departments of Medicine, Pediatrics, and Microbiology & Immunology, Weill Cornell Medical Center of Cornell University, New York, NY USA

**Keywords:** ^18^F-FDG PET/CT, Experimental osteomyelitis, *Staphylococcus aureus*, Daptomycin

## Abstract

**Background:**

18-Fluoro-2-deoxy-d-glucose positron emission tomography combined with computed tomography (^18^F-FDG PET/CT) scan is useful for diagnosis of osteoarticular infections. Whether ^18^F-FDG PET/CT scanning may be used for therapeutic monitoring is not clear. The objective of this study was to develop ^18^F-FDG PET/CT scanning for monitoring therapeutic response to antimicrobials in experimental *Staphylococcus aureus* osteomyelitis.

**Methods:**

A total of 22 rabbits were studied. In 20 animals, the right tibia was inoculated intraoperatively with *S. aureus*. Two control animals were inoculated with normal saline. A needle was placed in the tibia as a foreign body. Infection was allowed to develop for 21 days when ^18^F-FDG PET/CT was performed, the needle was removed, and bone specimens were cultured to confirm infection. Antimicrobial therapy with daptomycin was initiated in all successfully infected animals for 1, 3, or 6 weeks. Following completion of treatment, a second ^18^F-FDG PET/CT was performed, animals were euthanized, and infected tibias were harvested for quantitative cultures and histology. A positive scan was defined as ^18^F-FDG signal activity greater in the infected tibia than that of the contralateral non-infected control tibia. Therapeutic response was measured by the change of ^18^F-FDG signal activity in the infected tibia.

**Results:**

All successfully infected animals (*n* = 14), with microbiologically and/or histologically confirmed osteomyelitis, had positive ^18^F-FDG PET/CT scans, while the two control animals had negative scans despite the presence of the foreign body [mean maximum standardized uptake value (SUVmax) (±SD) values 2.96 (±0.80) vs. 1 (±1.10), respectively, *P* = 0.04]. In the 14 successfully infected animals, the mean SUVmax was significantly higher in the infected compared to the uninfected tibia (*P* < 0.0001). A SUVmax of 1.4, when used as a cutoff for infection, yielded a diagnostic accuracy of 93 %. At the end of treatment, successfully treated animals and saline controls had a negative ^18^F-FDG PET/CT scan (*n* = 4), while animals with persistent infection despite treatment (*n* = 12) had a positive ^18^F-FDG PET/CT scan (SUVmax 1.0–3.0) (*p* < 0.001). SUVmax values were significantly reduced after 42 days of treatment from 3.15 ± 0.5 (day 7) to 1.71 ± 0.37 (day 42) (*p* = 0.05).

**Conclusions:**

^18^F-FDG PET/CT scan is a sensitive and specific tool in therapeutic monitoring of experimental foreign-body osteomyelitis.

## Introduction

Prosthetic joint infection (PJI) remains one of the most dreadful complications of prosthetic joint implantation. As clinical signs and symptoms of PJI are usually non-specific, indolent, and vague, the diagnosis can be difficult even when accompanied by serologic, conventional radiographic, and microbiologic diagnostic tests [1, 2]. Plain radiographs are not helpful as they lack both sensitivity and specificity [3]. Computed tomography (CT) and magnetic resonance imaging (MRI) are limited by hardware-induced artifacts [1–3]. Functional imaging modalities have been extensively studied and applied for the diagnosis of PJI [4]. Yet, their diagnostic performance is not satisfactory and their routine use is not recommended [5]. Fluorine-18-fluoro-2-deoxy-d-glucose positron emission tomography combined with computed tomography (^18^F-FDG PET/CT) scan is a cutting-edge functional imaging as it provides images with higher resolution with concomitant anatomical information. ^18^F-FDG PET/CT has been reported in as a diagnostic imaging modality tor detection of PJIs [2, 4, 6–13]; however, little is known about its potential role for monitoring of response to antimicrobial therapy of osteomyelitis and PJI.

The management of PJI often necessitates a two-stage exchange procedure, i.e., removal of the infected prosthesis, prolonged courses of antimicrobial therapy, and re-implantation after the clearance of infection [14, 15]. One unresolved issue is the optimal time for re-implantation, because if infection reoccurs after a two-stage exchange has been accomplished, the success rate with a second two-stage exchange attempt may be lower than with the first attempt [15]. Currently, there are no diagnostic tools for monitoring the response to antimicrobial therapy and reliably exclude persistent joint infection, since clinical assessment, conventional imaging modalities, and laboratory tests may not be accurate [15]. ^18^F-FDG PET/CT scanning has the potential to provide objective parameters in the form of imaging and standardized uptake value (SUV) signal strength for the monitoring of therapeutic response in osteomyelitis.

We therefore studied ^18^F-FDG PET/CT scanning for therapeutic monitoring in a rabbit model of *Staphylococcus aureus* experimental foreign-body osteomyelitis. The experiments were designed to assess ^18^F-FDG PET/CT scan monitoring the success of antimicrobial therapy in eradicating bone infection after removal of the foreign body. To our knowledge, this is the first study of ^18^F-FDG PET/CT scanning in therapeutic monitoring of systemic antibacterial treatment of experimental *S. aureus* osteomyelitis.

## Methods

The study was performed in the animal facility of the Center for Experimental Surgery of the Biomedical Research Foundation of the Academy of Athens. The facility is registered as a “breeding” and “experimental” facility according to the Greek Presidential Decree 160/91, on the protection of animal used for scientific purposes. The Veterinary Service of the Prefecture of Athens approved the protocol.

### Study animals

A total of 22 male New Zealand white rabbits at the age of 4 months old and a body weight between 3.0 and 3.5 kg were used in this study. The animals were purchased from a registered rabbit farm (Farma Trompetas, Megara, Attiki, Greece) and reported to be free of bacterial and protozoal pathogens.

Each rabbit was housed individually in a single cage in a stainless steel rabbit cage rack (Tecniplast, Buguggiate, Italy) in a room temperature of 21 ± 2 °C, a relative humidity of 50 ± 5 %, and with a 12:12-h light-to-dark cycle. The rabbits were fed *ad libitum* with a prepackaged certified fiber rabbit chow (12C, Pezzulo, Italy) and had unlimited access to water via an automatic watering system. Before surgery, all rabbits were acclimated to their new environment for a period of 5 days.

### Study protocol

**Fig. 1:**
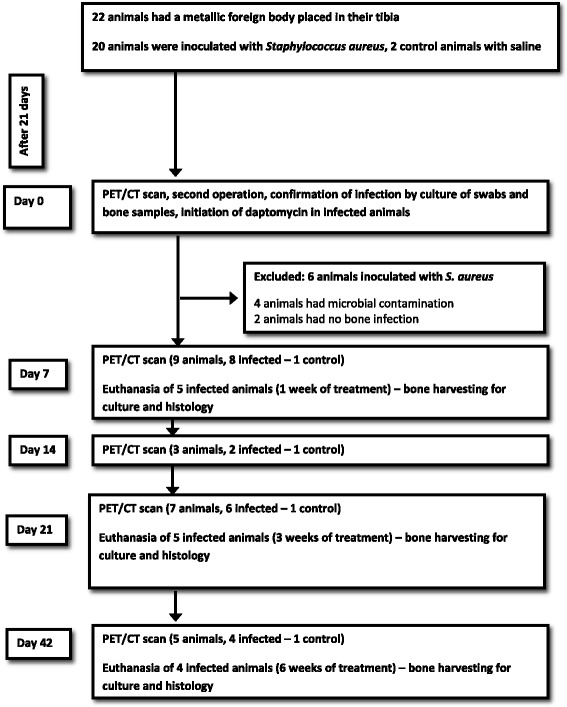
Flow chart of our animal model of experimental foreign-body osteomyelitis

The animal model and experimental design recapitulate the management of human PJI, including diagnosis of infection, removal of infected prosthesis, prolonged treatment with antibacterial agents, and assessment for clearance of infection before re-implantation (Fig. 1). A modified version of the tibial osteomyelitis model of Norden [16, 17] was used, in which the medullary cavity of the tibia was inoculated with methicillin-susceptible *S. aureus*, and a 25-gauge needle was inserted into the tibia and served as a foreign body, in both infected animals and controls. Control animals were inoculated with normal saline. Infection was allowed to develop for 21 days, and then an ^18^F-FDG PET/CT was performed, and blood cultures were taken. At this time point, all the animals, infected and controls, underwent a second stage surgery where the defect area was exposed, the needle was removed, the wound was debrided, and swab specimens and bone fragments taken with a curette were cultured to confirm the presence of infection. A bone culture yielding >10^4^ colony-forming unit (cfu)/ml of the initially inoculated *S. aureus* strain was interpreted as established osteomyelitis.

Immediately after the second operation, antimicrobial treatment with daptomycin (4 mg/kg every 12 h subcutaneous) was initiated in all successfully infected animals. This dosage provides comparable pharmacokinetics in rabbits and humans and has been previously studied in experimental non-prosthetic *S. aureus* osteomyelitis, where it achieved bone levels exceeding that of plasma [18–20]. The rabbits were allocated to receive treatment for 1, 3, or 6 weeks, in order to assess the correlation of ^18^F-FDG uptake with the gradual resolution of infection at different treatment time points. Following completion of the treatment, animals underwent a second evaluation including ^18^F-FDG PET/CT and blood cultures. Then, rabbits were euthanized and infected tibias were harvested for quantitative microbiological cultures and histology. A bone infection was considered as cured when at the end of treatment bone cultures were negative for pathogens and histology was negative for findings of infection.

### Bacterial inoculum

The methicillin-susceptible *S. aureus* ATCC 29213 strain (American Type Culture collection, Manassas, VA, USA) was used for the preparation of the bacterial inoculum. The isolate was grown overnight in Tryptic Soy Broth (TSB) (Bioprepare, 19001, Keratea, Greece), washed three times in ice-cold sterile buffered salt solution, and adjusted to a bacterial suspension equivalent to 0.5 of the McFarland scale, measured at 600 nm (approximately 1.5 × 10^8^ cfu/ml).

### Technique of infection

The animals were anesthetized through the administration of an intra-muscular injection of ketamine (35 mg/kg, Imalgene 1000; Merial, Lyon, France) and xylazine (5 mg/kg Rompun; Bayer Animal Health GmbH, Leverkusen, Germany). The animals were intubated and connected to a veterinary ventilator (MDS Matrx Model 2000; Hallowell EMC, Pittsfield, MA, USA). Anesthesia was maintained with the administration of 1 minimum alveolar concentration (MAC) isoflurane (1.7–2 %, Forenium, Abbott, Abbot Park, IL, USA). Electrocardiography (DASH 2000 Pro, GE Healthcare, Milwaukee, WI, USA), vital signs, and oxygen saturation were monitored throughout the surgical procedure through appropriate electrodes, a pulse oximeter, and a rectal temperature probe (9-F, Mon-a-Therm; Mallinckrodt, St Louis, MI, USA).

Surgery was performed using standard aseptic techniques with one operator performing all procedures. A 2-cm incision was made in the skin 2.5 cm below the right knee joint, which exposed the medial cortex of the tibial metaphysis. A cortical defect was made with a 2-mm drill bit, and 0.1 ml (approximately 1.5 × 10^7^ cfu) of the prepared bacterial suspension was instilled. A 25-gauge needle was placed through the hole and served as a foreign body, and the hub of the needle was broken off. Finally, the drilling hole was plugged with sterile bone wax to prevent bacterial leakage. The surgical field was lavaged with 50 ml of sterile saline water before wound closure in layers. The wound was cleaned using sterile saline water, and no sealing wound coverage was used. In control animals, a cortical defect of equal size was drilled but 0.1 ml of sterile saline water was inoculated. Further steps were similar as in the infectious animal group, up to wound closure and cleaning with sterile saline water. Then, neomycin sulfate spray (Pulvo 47, Abott Laboratories, Hellas) was applied (manufacturer details), and a single dose of cefuroxime sodium (25 mg/kg, Zinacef, GlaxoSmithKline, UK) was given IM postoperatively. Animals returned to their cages and given carprofen (4 mg/kg, Rimadyl, Pfizer, UK) analgesia as needed every 12 h.

### ^18^F-FDG PET/CT imaging

^18^F-FDG PET/CT scans were obtained for each animal at day 21 after the induction of osteomyelitis, as well as at the end of treatment, just before euthanasia (Fig. 1). The animals were not fed for at least 6 h prior to tracer injection. Each animal was scanned 30 min after the intravenous administration of 74 MBq (2 mCi) of ^18^F-FDG in the ear artery. Scanning was performed using a combined ^18^F-FDG PET/CT scanner (Siemens Biograph 6, high-resolution PET/CT, Knoxville, TN, USA). The acquisition time was 10 min per bed position for a total of two bed positions (20 min total acquisition time). The CT data were used for attenuation correction, and images were reconstructed using a standard ordered-subset expectation maximization (OSEM) algorithm. CT images were acquired with 85 mA, 130 KV, axial slice thickness of 3.0 mm, and table feed rotation of 10.0 mm per rotation. Data were corrected for dead time, decay, and photon attenuation. The image reconstruction matrix was 256 × 256. Fusion of the PET and CT images was performed. Every ^18^F-FDG PET/CT scan was assessed by visual inspection by two experienced nuclear medicine physicians blinded to the presence or not of osteomyelitis or to the timing of the scan as far as treatment was concerned. In case of disagreement, a third experienced nuclear medicine physician assessed the images and a consensus was achieved. An ^18^F-FDG PET/CT scan was considered as positive when visual inspection showed increased activity in the area of the inoculation compared to the adjacent normal bone or to the contralateral tibia. Measurements of tracer uptake were expressed as the maximum standardized uptake value (SUVmax) in the region of operated left tibia. Comparative measurements were made for each animal (expressed as SUVmax) in the corresponding region of the contralateral intact right tibia.

### Microbiological analysis

Tissue specimens were collected in TSB (Bioprepare, Keratea, Greece) and homogenized using a Potter-Elvehjem PTFE tissue grinder (Sigma-Aldrich GmbH, 82024, Germany). Swabs were diluted in 0.5 ml TSB (Bioprepare, Keratea, Greece) and vortexed for 10 s. Quantitative cultures from the tissue homogenizations and the swab dilutions were performed on Columbia agar plates supplemented with 5 % horse blood (Bioprepare, Keratea, Greece) and incubated for 48 h in 5 % CO_2_ conditions. Any bacterial growth that was detected was sub-cultured and identified as the original inoculum based on common phenotypic and susceptibility testing patterns.

### Histopathology

Separate cancellous bone particles harvested on sacrifice were fixed in neutral buffered formalin, embedded in paraffin wax, and sectioned and stained with hematoxylin and eosin. Tissues were examined for the presence of abscesses, type of inflammatory cells, and osseous injury.

### Statistical analysis

In order to determine whether the mean SUVmax was different between the left (infected) and right (normal) upper tibial metaphysis of the animals, the null hypothesis was that the mean SUVmax was the same between the two sides. Differences in SUV signals between normal and infected areas of the same animal were analyzed paired *t* test. The receiver operating characteristic (ROC) analysis was used to define the cutoff SUVmax value for infection. Differences in therapeutic response as measured by the intensity of SUV signal were assessed between days 7 and 42 unpaired by *t* test. Comparisons of differences in proportions were analyzed by Fisher’s exact test. Values were expressed as mean ± SD, and statistical significance was defined as *P* ≤ 0.05.

## Results

### Establishment of infection and imaging findings on day 21 after inoculation

A total of 22 animals were studied; 20 animals received an intramedullary inoculum of *S. aureus* suspension, and two control animals received sterile saline. On day 21 after inoculation, all 22 rabbits underwent an ^18^F-FDG PET/CT scan followed by a second stage surgery, where swab specimens and bone samples were taken for laboratory studies. Swab and bone cultures established the presence of osteomyelitis, due to the inoculated strain of *S. aureus* in 14 animals, while the inoculated pathogen was not isolated in four animals (two controls and two animals inoculated with *S. aureus*). In four animals, the bone cultures grew pathogens other than the inoculated *S. aureus* strain, notably *Staphylococcus epidermidis*, probably due to contamination. All animals with failure to establish infection (*n* = 2) or contamination (*n* = 4) were excluded from the study resulting in a control group of 2 animals and an infection group of 14 animals (Fig. 1). In all animals, blood cultures taken on day 21 were negative for pathogens.

**Fig. 2:**
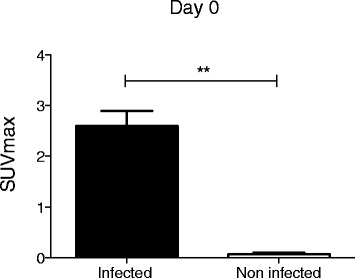
SUVmax values at day 21 after inoculation of *S. aureus* (*n* = 14) or normal saline (*n* = 2) in the tibia of the animals (**P < 0.05)

All 14 animals with established infection had a positive ^18^F-FDG PET/CT scan with SUVmax values ranging from 0.9 to 4.8 (Tables 1 and 2). The two animals in the control group had a negative ^18^F-FDG PET/CT scan, despite the presence of the foreign body. The mean SUVmax ratios were 1 (SD 1.10) and 2.96 (SD 0.80) for the control and osteomyelitis animal group, respectively (*P* = 0.04) (Fig. 2). In the 14 successfully infected animals, the mean SUVmax was found to be significantly higher in the infected tibia compared to the contralateral non-infected tibial metaphysis (*t* value 5.59, *P* value of 9 × 10^−5^) and the null hypothesis was rejected.

**Table 1:** Bone infection, treatment, and ^18^F-FDG PET/CT scan findings in rabbit model of *S. aureus* osteomyelitis

Animal number	Day 21	Day 0		Tx	Duration of treatment (weeks)	Day 7 of treatment/follow-up	Day 14 of treatment/follow-up	Day 21 of treatment/follow-up	Day 42 of treatment/follow-up	Bone infection at the end of treatment/follow-up
	Inoculum	Bone infection	PET/CT (SUVmax)			PET/CT (SUVmax)	PET/CT (SUVmax)	PET/CT (SUVmax)	PET/CT (SUVmax)	
706	Saline	No	Neg	No	–	Neg	Neg	–	–	No
693	Saline	No	Neg	No	–	–	–	Neg	Neg	No
700	*S. aureus*	No	Neg	No	–	–	–	–	–	–
701	*S. aureus*	No	Neg	No	–	–	–	–	–	–
698	*S. aureus*	Yes	Pos (2.6)	Yes	1	Pos (3.9)	–	–	–	Yes
699	*S. aureus*	Yes	Pos (4.1)	Yes	1	Pos (4.8)	–	–	–	Yes
702	*S. aureus*	Yes	Pos (3.2)	Yes	1	Pos (5.3)	–	–	–	Yes
703	*S. aureus*	Yes	Pos (2.6)	Yes	1	Pos (2.3)	–	–	–	Yes
707	*S. aureus*	Yes	Pos (1.7)	Yes	1	Pos (2.0)	–	–	–	Yes
695	*S. aureus*	Yes	Pos (1.8)	Yes	3	–	–	Pos (3.9)	–	Yes
694	*S. aureus*	Yes	Pos (3.5)	Yes	3	–	–	Pos (1.8)	–	Yes
692	*S. aureus*	Yes	Pos (1.4)	Yes	3	Pos (1.5)	Pos (1.6)	Neg (0.8)	–	No
704	*S. aureus*	Yes	Pos (1.9)	Yes	3	Pos (3.6)	–	Pos (3.8)	–	Yes
705	*S. aureus*	Yes	Pos (2.0)	Yes	3	Pos (1.8)	–	Pos (1.9)	–	Yes
708	*S. aureus*	Yes	Pos (3.4)	Yes	6	–	–	–	Pos (1.6)	Yes
709	*S. aureus*	Yes	Pos (2.5)	Yes	6	–	–	–	Pos (1.8)	Yes
716	*S. aureus*	Yes	Pos (4.8)	Yes	6	–	–	–	Neg (0.8)	No
718	*S. aureus*	Yes	Pos (0.9)	Yes	6	–	Pos (1.7)	Pos (2.8)	Pos (2.6)	Yes

*Tx* treatment, *SUVmax* maximum standardized uptake value, *Pos* positive, *Neg* negative

**Table 2:** SUVmax values from PET images in 14 successfully infected animals with *S. aureus* osteomyelitis

^18^F-FDG PET/CT scan	Number of animals	Average SUVmax		Maximum SUVmax		Minimum SUVmax	
		Left tibia	Right tibia	Left tibia	Right tibia	Left tibia	Right tibia
21 days post-inoculation, no treatment	14	2.6	1.1	4.8	1.4	0.9	0.7
7 days of treatment	8	3.2	1.2	5.3	1.4	1.5	0.9
14 days of treatment	2	1.6	1.0	1.7	1.1	1.6	0.8
21 days of treatment	6	2.5	1.0	3.9	1.4	0.8	0.8
42 days of treatment	4	1.7	0.8	2.6	0.8	0.8	0.8

*SUVmax* maximum standardized uptake value

**Fig. 3:**
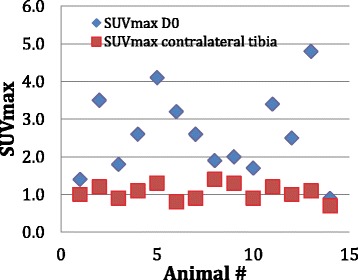
The distribution of the SUVmax values for the infected (*DO*) and the normal contralateral tibia (*right*) of the 14 animals calculated from the first ^18^F-FDG PET/CT scan in the series

Figure 3 shows the distribution of the SUVmax values for the infected (left) and the normal site (right) of the 14 animals calculated from the first ^18^F-FDG PET/CT scan in the series. ROC analysis showed an SUVmax value of 1.4 as a cutoff value for infection with an accuracy of 93 % as well as a clear demarcation between infected and normal sites.

### Antimicrobial treatment and follow-up studies (Table 1)

**Fig. 4:**
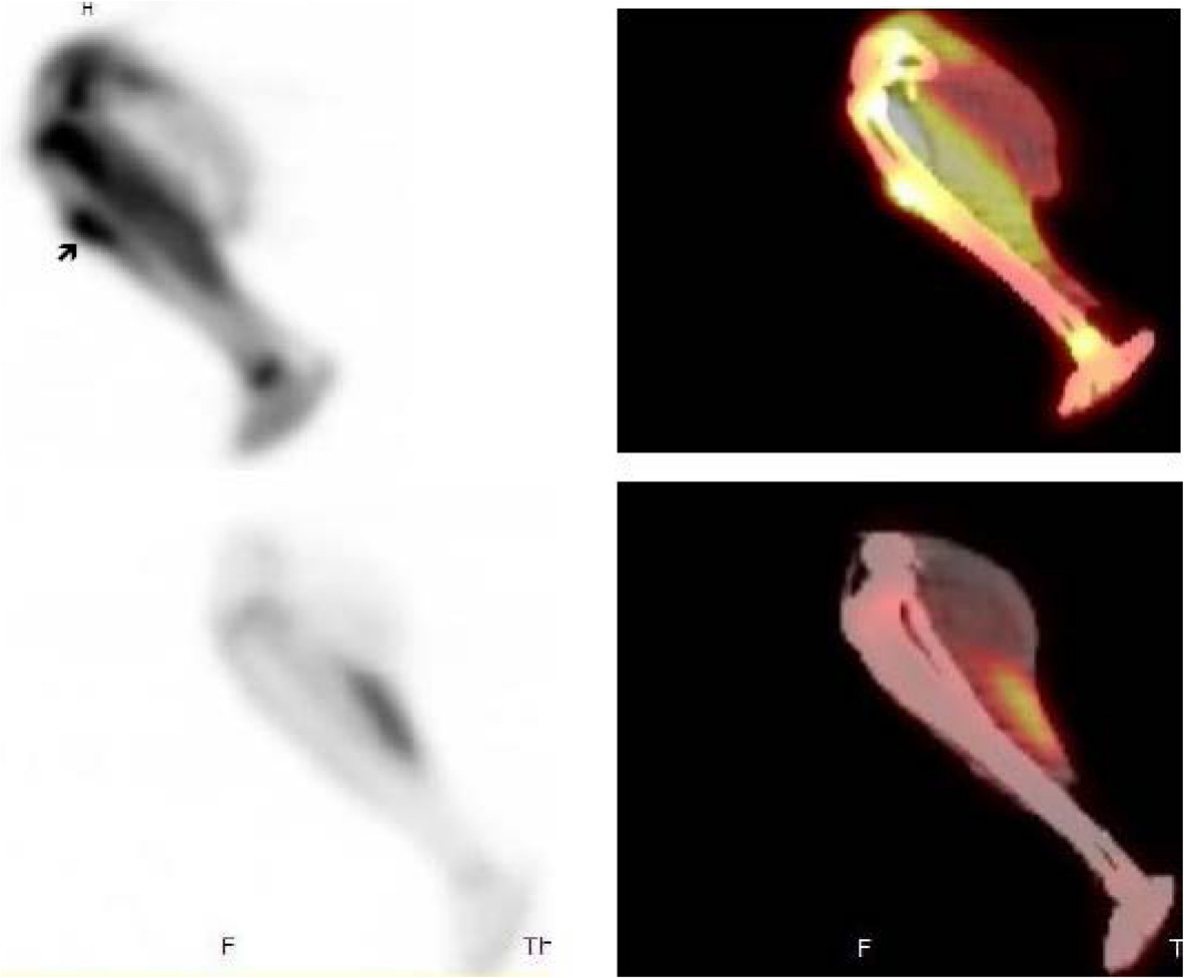
^18^F-FDG PET/CT scan images of a successfully treated rabbit with experimental tibial staphylococcal osteomyelitis. Sagittal images of the tibia of a rabbit demonstrating (*top*) increased tracer uptake (SUVmax = 4.8) at the area of osteomyelitis in the upper third of the tibia extending to the adjacent soft tissues (*arrow*) before the initiation of treatment and (*bottom*) physiologic tracer activity in the tibia of the same rabbit after 6 weeks of treatment with daptomycin. The animal was euthanized immediately after the ^18^F-FDG PET/CT scan, and bone cultures and histology were negative for infection. Note: On the *left* are the PET images and on the *right* the fused PET/CT images

**Fig. 5:**
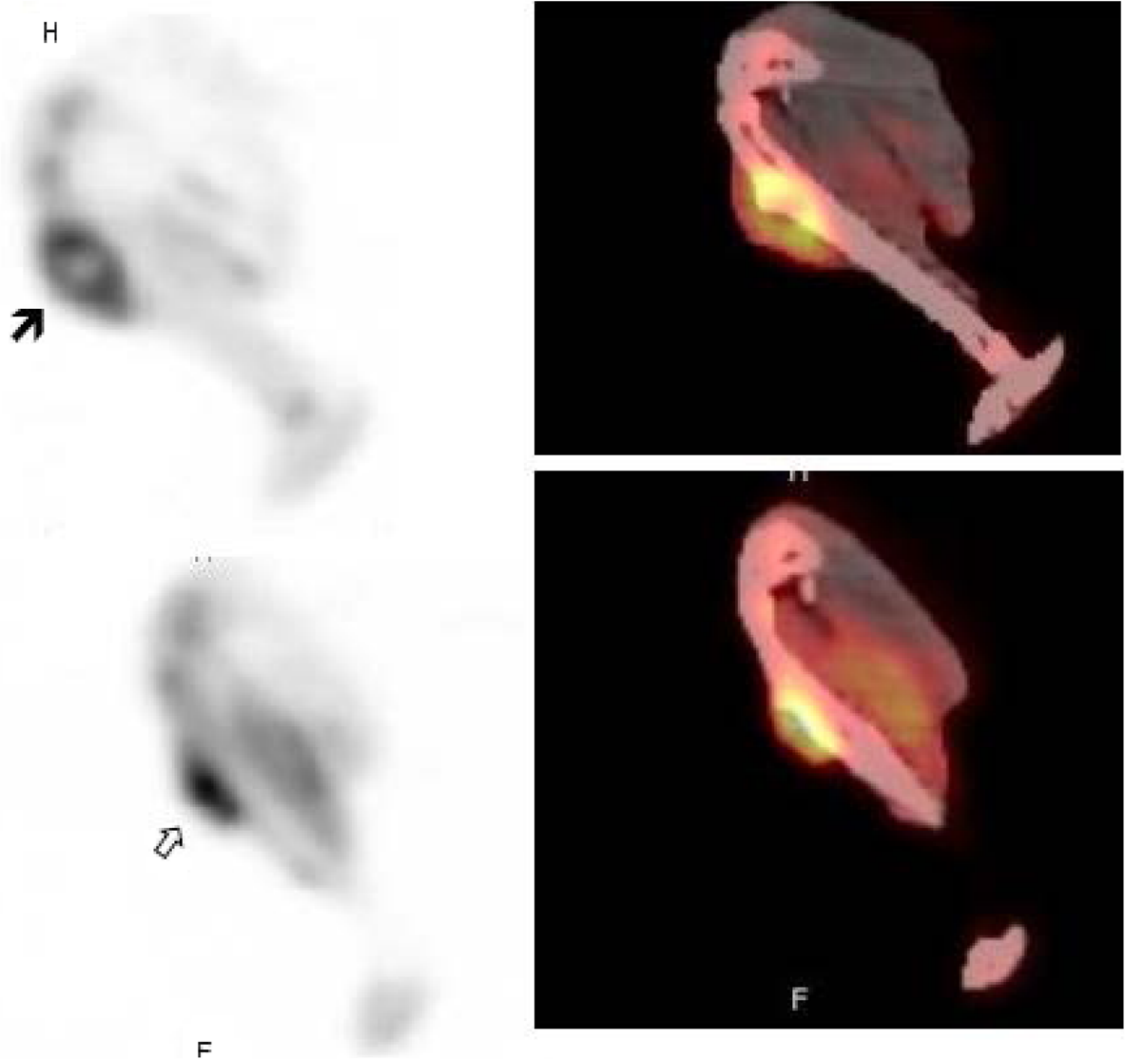
^18^F-FDG PET/CT scan images of an unsuccessfully treated animal with experimental staphylococcal tibial osteomyelitis. Sagittal images of the tibia of a rabbit demonstrating (*top*) increased tracer uptake (SUVmax = 3.6) with central photopenia at the area of osteomyelitis in the upper third of the tibia extending to the adjacent soft tissues (*dense arrow*) after 1 week of treatment and (*bottom*) persistent tracer activity (SUVmax = 3.8) in the tibia of the same rabbit (*hollow arrow*) after 3 weeks of treatment. The animal was euthanized immediately after the second ^18^F-FDG PET/CT scan, and both bone cultures and histology showed active infection. Note: On the *left* are the PET images and on the *right* the fused PET/CT images

All animals with established osteomyelitis (*n* = 14) were treated with daptomycin for 2 weeks (five animals), 3 weeks (five animals), or 6 weeks (four animals). All animals survived until the end of the treatment period, when a second ^18^F-FDG PET/CT scan was performed. A positive ^18^F-FDG PET/CT scan was observed in all five animals treated for 1 week; four of the five animals treated for 3 weeks; and three of the four animals treated for 6 weeks (Figs. 4 and 5). In total, at the end of the treatment, 12 animals had a positive ^18^F-FDG PET/CT scan (SUVmax 1.0–3.0) and 2 animals had a negative ^18^F-FDG PET/CT scan.

### Effect of treatment on SUVmax values

**Fig. 6:**
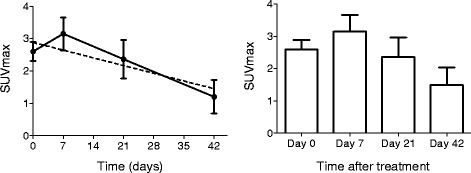
Effect of antimicrobial treatment on the SUVmax values. Initiation of antimicrobial treatment resulted in statistically significant (*p* = 0.03) reduction in the SUVmax values (*left panel*). The reduction was prominent at 42 days of treatment (*right panel*)

Tables 1 and 2 demonstrate the SUVmax before and after treatment. Three weeks after the inoculation of *S. aureus* and before the initiation of antimicrobial treatment, the mean SUVmax value of the 14 animals that were successfully infected was 2.6. The mean SUVmax value was 3.66 in eight animals treated for 1 week, 2.85 in six animals treated for 3 weeks, and 2.0 in four animals treated for 6 weeks (*P* < 0.05) (Fig. 6). At the end of the treatment, successfully treated animals and saline controls had a negative ^18^F-FDG PET/CT scan (*n* = 4), while animals with persistent infection despite treatment (*n* = 12) had a positive ^18^F-FDG PET/CT scan (SUVmax 1.0–3.0) (*p* < 0.001).

### Correlation between SUVmax values and quantitative cultures

After the ^18^F-FDG PET/CT scans, animals were euthanized, infected tibias were harvested, and swabs, as well as specimens of cancellous bone, were sampled under sterile conditions for laboratory studies. The inoculated strain of *S. aureus* was cultured at >10^4^ cfu/ml or cfu/gram of bone, from all harvested bones of the 12 animals with a positive ^18^F-FDG PET/CT scan.

Histology revealed active osteomyelitis in all specimens with positive cultures ≥10^4^ cfu/ml. These findings were interpreted as treatment failure. In contrast, the two successfully treated animals with negative ^18^F-FDG PET/CT scans and the two saline non-infected controls had negative bone and/or swab cultures and no histological evidence of active infection.

## Discussion

This study found that ^18^F-FDG PET/CT scan is a sensitive and specific tool in therapeutic monitoring of experimental foreign-body osteomyelitis. The study also found that ^18^F-FDG PET/CT scan accurately distinguishes between a bone with a metal implant and infection from a bone with a metal implant but without infection. All successfully infected animals, with microbiologically and/or histologically confirmed foreign-body osteomyelitis, had positive ^18^F-FDG PET/CT scan, while all animals without infection had negative ^18^F-FDG PET/CT scan, despite the presence of the foreign body. In all successfully infected animals, ^18^F-FDG PET/CT scan demonstrated increased tracer uptake in the infected area, compared to the control group (*P* < 0.05). In addition, compared to the contralateral not infected tibia, the SUVmax was significantly higher in the infected one (*P* < 0.05). We also found that a SUVmax cutoff value of 1.4 was able to differentiate foreign-body osteomyelitis from normal bone, with an accuracy of 93 %.

The rabbit model of *S. aureus* osteomyelitis has been used to explore the diagnostic efficacy of ^18^F-FDG PET scan. Lankier et al. showed that foreign-body-associated infection in the rabbit tibia caused by *S. epidermidis* results in lower ^18^F-FDG uptake than pyogenic *S. aureus* infections [21]. Researchers have shown by using a rabbit model of *S. aureus* osteomyelitis that bone healing was associated with a temporary increase in ^18^F-FDG uptake at 3 weeks, but it returned to normal by 6 weeks [22]. In contrast, localized osteomyelitis resulted in a significantly higher, continuous uptake of ^18^F-FDG. Similar results have been reported in a clinical study [9]. In clinical practice, ^18^F-FDG PET has demonstrated promising results for diagnosis of osteomyelitis. Nevertheless, its role in diagnosing PJI is less documented, as in a recent review sensitivity varied widely from 28 to 91 % and specificity from 9 to 97 % [6]. The discrepancy between the promising results in animal models and the wide variation in ^18^F-FDG PET/CT performance in the diagnosis of PJI in clinical studies might reflect the lack of criteria to interpret the ^18^F-FDG PET/CT scan findings suggestive of infection.

The main objective of our study was to establish the accuracy of ^18^F-FDG PET/CT scan for monitoring the therapeutic response of bacterial osteomyelitis to antimicrobial therapy, after removing the foreign body. We have shown in our animal model that the SUVmax values were significantly lower after 42 days of treatment. Additionally, all 12 animals with documented persistent infection after completion of antimicrobial therapy had a positive ^18^F-FDG PET/CT scan, while in 2 animals where the infection had been eradicated, the ^18^F-FDG PET/CT scans were negative. The clinical implications of these findings are obvious as in clinical practice, the existing diagnostic modalities, such as clinical assessment, imaging findings, or serology, cannot accurately exclude persistent joint infection after resection arthroplasty [14, 15]. Re-implantation without previous clearance of infection might compromise the efficacy of the two-stage exchange procedure for the treatment of PJI. Our experimental data suggest that in clinical practice, ^18^F-FDG PET/CT scan might have a role in excluding persisting infection before re-implantation.

Our study has several limitations. One isolate of *S. aureus* was used. While other isolates of *S. aureus* may have a different pro-inflammatory response, this is unlikely. A baseline and end of therapy scan were performed with no intervening scans; however, the study design does allow for understanding different groups of animals at different time points. A single dosage of daptomycin was used. Although this study was not designed to determine the optimal dosage and duration of daptomycin for treatment of foreign-body osteomyelitis, it establishes a conceptual framework by which a larger study may be conducted. The use of multiple experimental animals, sacrificed at pre-defined time points, and the direct documentation or exclusion of infection by means of both bone culture and histology establishes a correlation between therapeutic response and ^18^F-FDG PET/CT imaging technology.

## Conclusions

In summary, ^18^F-FDG PET/CT imaging technology is a sensitive and specific tool in the diagnosis of experimental foreign-body osteomyelitis and in monitoring the therapeutic response of experimental osteomyelitis after removal of the foreign body. These data can be used in the development of a prospective clinical trial in the use in the diagnosis and therapeutic monitoring of human PJI with the use of ^18^F-FDG PET/CT scan.

## Abbreviations

^18^F-FDG PET/CT: fluorine-18 fluoro-2-deoxy-d-glucose positron emission tomography combined with computed tomography
MRI: magnetic resonance imaging
PJI: prosthetic joint infection
SUVmax: maximum standardized uptake value
